# The efficacy and safety of S-1-based regimens in the first-line treatment of advanced gastric cancer: a systematic review and meta-analysis

**DOI:** 10.1007/s10120-015-0587-8

**Published:** 2016-01-11

**Authors:** Emil ter Veer, Nadia Haj Mohammad, Paul Lodder, Lok Lam Ngai, Mary Samaan, Martijn G. H. van Oijen, Hanneke W. M. van Laarhoven

**Affiliations:** Department of Medical Oncology, Academic Medical Centre, University of Amsterdam, Meibergdreef 9, F4-224, 1105 AZ Amsterdam, The Netherlands; Department of Psychology, University of Amsterdam, Amsterdam, The Netherlands

**Keywords:** Advanced gastric cancer, S-1, Chemotherapy, Meta-analysis

## Abstract

**Background:**

S-1 is first-line therapy for advanced gastric cancer in Asia and is used with increased frequency in Western counties. We conducted a meta-analysis to investigate the efficacy and toxicity of S-1-based therapy compared with 5-fluorouracil (5-FU)/capecitabine-based therapy and S-1-based combination therapy compared with S-1 monotherapy.

**Methods:**

MEDLINE, Embase, the Cochrane Central Register of Controlled Trials, American Society of Clinical Oncology meeting abstracts, European Society for Medical Oncology meeting abstracts and ClinicalTrials.gov were searched for randomized clinical trials until May 2015. Data were extracted for overall survival (OS), progression-free-survival (PFS), objective response rate (ORR) and grade 1–2 and grade 3–4 adverse events. Stratified OS data for subgroups were extracted.

**Results:**

S-1 was not different from 5-FU (eight studies, *n* = 2788) in terms of OS [hazard ratio (HR) 0.93, 95 % confidence interval (CI) 0.85–1.01] and PFS (HR 0.87, 95 % CI 0.73–1.04), whereas ORR was higher (risk ratio 1.43, 95 % CI 1.05–1.96). There was no subgroup difference in efficacy among Asian and Western patients, but in Western patients S-1 was associated with a lower rate of febrile neutropenia, toxicity-related deaths and grade 3–4 stomatitis and mucositis compared with 5-FU. S-1 showed no difference in efficacy compared with capecitabine (three studies, *n* = 329), but was associated with a lower rate of grade 3–4 neutropenia and grade 1–2 hand–foot syndrome. S-1-combination therapy was superior to S-1 monotherapy (eight studies, *n* = 1808) in terms of OS (HR 0.76, 95 % CI 0.65–0.90), PFS (HR 0.68, 95 % CI 0.56–0.82) and ORR (risk ratio 1.20, 95 % CI 1.04–1.38) but was more toxic. Survival benefit of S-1 combination therapy over S-1 monotherapy was most pronounced in patients with non-measurable disease, diffuse-type histological features and peritoneal metastasis.

**Conclusions:**

S-1 is effective and tolerable as first-line therapy for advanced gastric cancer in both Asian and Western countries.

**Electronic supplementary material:**

The online version of this article (doi:10.1007/s10120-015-0587-8) contains supplementary material, which is available to authorized users.

## Introduction

Fluoropyrimidines are the backbone of first-line therapy for advanced gastric cancer [1, 2]. The novel fluoropyrimidine S-1 has quickly become the standard of care in Asia, but there is uncertainty about the role of S-1 in Western countries. Although S-1 is used with increasing frequency in Western countries, it has not fully replaced 5-fluorouracil (5-FU) and capecitabine. Meta-analyses have shown a marginally significant prolonged survival time and higher response rates for S-1 therapy compared with 5-FU therapy [3–5] but not for S-1 therapy compared with capecitabine therapy [6–9]. However, some of these reviews included retrospective studies, which may lead to bias of the overall effect observed or did not incorporate the newest evidence in this field [10–15]. For example, in addition to the FLAGS trial [16], which was conducted in Western countries, the recently presented DIGEST trial [11] can also shed light on the role of S-1 therapy in Western patients.

The use of doublets of cytotoxic agents versus singlets is associated with prolonged survival [17] and therefore S-1-based combination therapy versus S-1 monotherapy has been investigated in several large trials in Asia. Previous meta-analyses have indicated that combination therapy significantly prolonged survival over monotherapy, but generally combination therapy was more toxic [18, 19]. However, the final results of four randomized studies, including the pivotal START trial, which was the first phase III trial to compare S-1 combined with a taxane with S-1 alone, were not included in these reviews [12, 13, 20, 21]. Moreover, it is also still an open question if there are predictive factors to define which patient subgroups will benefit most from S-1 combination therapy compared with S-1 monotherapy.

Therefore, the objectives of our study were to systematically review all available literature on randomized clinical trials to investigate the efficacy and toxicity by means of meta-analysis of S-1-based therapy compared with 5-FU- and capecitabine-based therapy and of S-1-based combination therapy compared with S-1 monotherapy.

## Methods

### Study protocol

The protocol of this review has been published in the international prospective register of systematic reviews (PROSPERO): http://www.crd.york.ac.uk/PROSPERO/display_record.asp?ID=CRD42014010654.

### Literature search

For the searching of the electronic databases [MEDLINE, Embase and Cochrane Central Register of Controlled Trials (CENTRAL)], a sensitive search strategy without date restriction was applied using the medical subject headings of ‘S-1’ and ‘gastric cancer’; thereafter, the results were filtered for clinical trials. ClinicalTrials.gov (http://www.clinicaltrials.gov) was searched for the term ‘S-1’ within the topic ‘stomach neoplasm’ and the results were filtered for phase II and phase III trials. In addition, all meeting abstracts from the American Society of Clinical Oncology and European Society for Medical Oncology up to May 2015 were searched via http://www.ascopubs.org/search and http://annonc.oxfordjournals.org/search, respectively, for the following terms: ‘S-1’ and ‘gastric’. The full search history is available in Document S1 in the electronic supplementary material. Two reviewers (E.t.V. and M.S.) reviewed the literature independently, and discrepancies were resolved by discussion with an arbiter (N.H.M.) until consensus was reached. This systematic review was performed according to the Preferred Reporting Items for Systematic Reviews and Meta-analyses (PRISMA) statement.

### Inclusion criteria

Studies had to meet the following eligibility criteria: (1) included patients with pathologically proven advanced gastric cancer (recurrent or unresectable disease); (2) first-line palliative (a) S-1-based therapy (monotherapy or doublet therapy) compared with 5-FU- or capecitabine-based chemotherapy (monotherapy or doublet therapy ) or (b) S-1-based combination chemotherapy compared with S-1 monotherapy; and (3) prospective phase II or phase III randomized controlled trials.

### Outcomes and data extraction

The primary efficacy outcome was overall survival (OS). To identify potential predictive factors for the efficacy of S-1 combination therapy compared with S-1 monotherapy, subgroup data were extracted for OS if possible. Secondary efficacy outcomes were progression-free survival (PFS) and overall response rate (ORR), defined as the sum of both partial and complete responses according to the Response Evaluation Criteria in Solid Tumors (RECIST). Tolerability outcomes comprised the incidence of adverse events (AEs) divided into mild toxicity (grade 1–2 AEs) and severe toxicity (grade 3–4 AEs). In all studies, AEs were scored according to the National Cancer Institute Common Toxicity Criteria (http://ctep.cancer.gov). Two reviewers (E.t.V. and N.H.M.) were involved in data extraction; discrepancies were resolved by discussion with an arbiter (L.N.) until consensus was reached.

### Study quality assessment

Two reviewers (E.t.V. and N.H.M.) independently examined the quality of all included studies using the Cochrane risk of bias tool (*Cochrane Handbook for Systematic Reviews of Interventions*, version 5.1.0) until consensus was reached. Studies with a high risk of bias were not included in the analysis. Since the primary outcome, OS, would not be influenced by the absence of a blinded imaging review, this item was not scored as unknown or high risk of bias for OS. Single-centre studies and studies without a published full article were rated as unclear risk of other possible bias. To assess the effect of study quality on the pooled estimate, sensitivity analyses were conducted by (1) omission of studies described in conference reports only and (2) omission of studies stepwise according to unknown risk of bias rating on one item, on two items and on three or more items.

### Statistical analysis

Pairwise meta-analyses using random-effect models were conducted with the Metagen R package [22] and Review Manager 5.3. For OS and PFS outcomes, hazard ratios (HRs) and 95 % confidence intervals (CIs) were extracted by the method described by Tierney et al. [23]. An HR less than 1 indicates a beneficial effect for the experimental arm, and an HR of 0.80 or less was considered clinically meaningful [24]. In addition, stratified HRs for OS in the patient subgroups were pooled with meta-analysis, and subgroup differences were statistically tested with chi-square tests. Risk ratios (RRs) were calculated for ORR (an RR greater than 1 indicates a beneficial effect for the experimental arm) and for event counts of grade 1–2 and grade 3–4 toxicity in both arms (an RR less than 1 indicates a beneficial effect for the experimental arm).

Statistical heterogeneity was tested with the Cochran Q test and quantified by the *I*^2^ index. Substantial heterogeneity (*I*^2^ ≥ 30 %) was explored by subgroup and sensitivity analyses. We tested for funnel plot asymmetry by regressing study outcomes on the standard error of the effect size [25]. All analyses were based on the intention-to-treat population of the included studies. All tests were performed two-sided, and a *P* value of less 0.05 was considered statistically significant.

## Results

### Literature search and study quality

Three hundred and fifty-four unique references were identified through our searching MEDLINE, Embase and CENTRAL until May 2015, from which 326 were excluded after abstract screening, because of ineligibility according to the criteria for this review. Of the 28 reports remaining for full-text screening, four studies were eligible to assess S-1-based versus 5-FU-based therapy [26–29], two studies were eligible to assess S-1-based versus capecitabine-based therapy [30, 31] ,and six studies were eligible to assess S-1 combination therapy versus S-1 monotherapy [21, 32–35]. Searching ClinicalTrials.gov and the American Society of Clinical Oncology and European Society for Medical Oncology libraries provided additional reports of four unpublished phase III studies [11, 12, 14, 15] and two phase II studies [10, 13]. The total number of studies included was 18 (Fig. S1).

There were no major differences in study and patient characteristics among the studies included (Table 1), although one study included patients with diffuse gastric cancer only [11]. For the primary outcome, seven studies were rated as low risk of bias [28–34], whereas 11 studies were rated as unclear risk of bias because of the lack of information on one item (three studies) [12, 21, 35] or two items (three studies) [20, 27] or abstract and insufficient information for risk of bias assessment (five studies) [10, 11, 13–15] (Fig. S2).

**Table 1:** Study and patient baseline characteristics

Study	Phase	Region	Centre	Enrolment	Arm	*N*	Men	Median age^a^ (range)
Ajani et al. [26]	III	Western countries	Multicentre	May 2005 to Mar 2007	S-1 + Cis	521	382 (73 %)	59 (18–83)
					5-FU + Cis	508	347 (68 %)	60 (20–85)
Ajani et al. [11]	III	Western countries	Multicentre	Apr 2011 to Feb 2014	S-1 + Cis	239	124 (52 %)	56 (25–86)
					5-FU + Cis	122	60 (49 %)	56 (27–83)
Boku et al. [29]	III	Japan	Multicentre	Nov 2000 to Jan 2006	S-1	234	175 (75 %)	64 (58–69)
					5-FU	234	176 (75 %)	64 (57–69)
Huang et al. [27]	II	China	Multicentre	Nov 2007 to Apr 2010	S-1 + PTX	119	89 (75 %)	56 (18–74)
					5-FU + PTX	110	76 (69 %)	54 (19–72)
Jin et al. [12]	III	China	Multicentre	Jul 2005 to Oct 2006	S-1 + Cis	74	55 (74 %)	57 (24–80)
					S-1	77	56 (73 %)	57 (32–82)
					5-FU + Cis	73	61 (84 %)	58 (33–77)
Kim et al. [31]	II	Korea	Multicentre	Mar 2008 to Sep 2009	S-1 + Ox	65	44 (68 %)	60 (28–77)
					Cap + Ox	64	45 (70 %)	61 (20–75)
Kobayashi et al. [10]	II	Japan	Multicentre	Nov 2011 to Jun 2013	S-1 + Cis	54	30 (55 %)	65 (44–74)
					Cap + Cis	55	45 (81 %)	65 (25–74)
Koizumi et al. [32]	III	Japan	Multicentre	Mar 2001 to Nov 2006	S-1 + Cis	148	108 (73 %)	62 (33–74)
					S-1	150	116 (71 %)	62 (28–74)
Koizumi et al. [20]	III	Japan and Korea	Multicentre	Sep 2005 to Sep 2008	S-1 + DTX	314	227 (72 %)	65 (23–79)
					S-1	321	229 (71 %)	65 (27–79)
Komatsu et al. [34]	II	Japan	Multicentre	Aug 2003 to Apr 2007	S-1 + IRI	48	34 (71 %)	70 (47–78)
					S-1	47	37 (79 %)	63 (24–76)
Lee et al. [30]	II	Korea	Multicentre	Oct 2004 to Apr 2006	S-1	45	37 (82 %)	71 (65–82)
					Cap	46	30 (65 %)	71 (66–78)
Lu et al. [21]	II	China	Single centre	Jan 2008 to Dec 2011	S-1 + Ox	47	34 (72 %)	63 (37–75)
					S-1	47	33 (70 %)	65 (34–74)
Narahara et al. [33]	III	Japan	Multicentre	Jun 2004 to Apr 2007	S-1 + IRI	155	110 (71 %)	63 (33–75)
					S-1	160	127 (79 %)	63 (27–75)
Nishikawa et al. [28]	II	Korea	Multicentre	Dec 2005 to Nov 2008	S-1 + PTX	77	53 (69 %)	67 (40–82)
					5-FU + PTX	80	60 (75 %)	67 (47–90)
Wang et al. [35]	II	China	Single centre	Jan 2009 to Dec 2011	S-1 + PTX	41	32 (78 %)	63 (35–74)
					S-1	41	30 (73 %)	61 (31–73)
Sawaki et al. [15]	III	Japan	Multicentre	May 2002 to Aug 2006	S-1	88	66 (75 %)	63 (32–77)
					5-FU + Lv	89	71 (80 %)	65 (44–77)
Xu et al. [14]	III	China	Multicentre	Sep 2008 to Dec 2011	S-1 + Cis	120	84 (70 %)	53 (25–76)
					5-FU + Cis	118	85 (73 %)	55 (21–76)
Yamaguchi et al. [13]	II	Japan	Multicentre	Oct 2011 to Dec 2012	S-1 + Cis	48	38 (79 %)	65
					S-1 + Lv	47	33 (70 %)	65
					Ox + S-1 + Lv	47	37 (79 %)	65

Study	ECOG PS ≥2	Metastatic	Regimen	2nd line	Median no. of cycles	Median OS (months)	Median PFS (months)
Ajani et al. [26]	0 (0 %)	497 (96 %)	S-1 50 mg/m^2^ days 1–21 + Cis 75 mg/m^2^ day 1 q4w	154 (30 %)	4	8.6	4.8
	0 (0 %)	488 (96 %)	5-FU 1000 mg/m^2^/24 h days 1–5 + Cis 100 mg/m^2^ day 1 q4w	169 (33 %)	4	7.9	5.5
Ajani et al. [11]	0 (1 %)	239 (100 %)	S-1 50 mg/m^2^ days 1–21 + Cis 75 mg/m^2^ day 1 q4w	NA	NA	7.5	4.4
	0 (0 %)	122 (100 %)	5-FU 800 mg/m^2^ days 1–5 + Cis 80 mg/m^2^ days1 q3w	NA	NA	6.6	3.9
Boku et al. [29]	3 (1 %)	234 (100 %)	S-1 80 mg/m^2^ days 1–28 q6w	173 (74 %)	NA	11.4	4.2
	3 (1 %)	234 (100 %)	5-FU 800 mg/m^2^ days 1–5 q4w	194 (83 %)	NA	10.8	2.9
Huang et al. [27]	Median KPS 80	112 (94 %)	S-1 80–120 mg/day days 1-14 + PTX 60 mg/m^2^ days 1, 8, and 15 q4w	NA	99 days (median exposure)	NA	153
	Median KPS 80	102 (93 %)	5-FU 500 mg/m^2^ days 1–5 + Lv 20 mg/m^2^ days 1–5 + PTX 60 mg/m^2^ days 1, 8, and 15 q4w	NA	77 days (median exposure)	NA	129
Jin et al. [12]	8 (11 %)	74 (100 %)	S-1 80 mg/m^2^ days 1–21 + Cis 60 mg/m^2^ day 8 q5w	NA	4.08^b^	14.2	NA
	12 (16 %)	77 (100 %)	S-1 80 mg/m^2^ days 1–28 q6w	NA	3.25^b^	8.8	NA
	10 (14 %)	73 (100 %)	5-FU 600 mg/m^2^ days 1–5 + Cis 20 mg/m^2^ days 1–5 q4w	41 (56 %)	2.77^b^	10.5	NA
Kim et al. [31]	0 (0 %)	47 (72 %)	S-1 80 mg/m^2^ days 1–14 + Ox 130 mg/m^2^ day 1 q3w	39 (60 %)	6	12.4	6.2^c^
	0 (0 %)	46 (72 %)	Cap 2000 mg/m^2^ days 1–14 + Ox 130 mg/m^2^ day 1 q3w	40 (62 %)	8	13.3	7.2^c^
Kobayashi et al. [10]	1 (2 %)	54 (100 %)	S-1 80 mg/m^2^ days 1–21 + Cis 60 mg/m^2^ day 8 q5w	NA	NA	8.3	3.6
	2 (4 %)	55 (100 %)	Cap 2000 mg/m^2^ days 1–14 + Cis 80 mg/m^2^ day 1 q3w	NA	NA	8.0	3.3
Koizumi et al. [32]	4 (3 %)	148 (100 %)	S-1 80–120 mg/day days 1–21 + Cis 60 mg/m^2^ day 8 q5w	110 (74 %)	4	13.0	6.0
	5 (3 %)	150 (100 %)	S-1 80–120 mg/day days 1–28 q6w	113 (75 %)	3	11.0	4.0
Koizumi et al. [20]	0 (0 %)	314 (100 %)	S-1 80–120 mg/day days 1-14 + DTX 40 mg/m^2^ day 1 q3w	219 (70 %)	NA	12.5	5.3
	0 (0 %)	321 (100 %)	S-1 80 mg/m^2^ days 1–28 q6w	244 (76 %)	NA	10.8	4.2
Komatsu et al. [34]	0 (0 %)	48 (100 %)	S-1 80–120 mg/m^2^ days 1-14 + IRI 75 mg/m^2^ days 1–15 q4w	NA	3	9.1	4.9^c^
	0 (0 %)	47 (100 %)	S-1 80–120 mg/m^2^ days 1–14 q4w	NA	2	12.3	3.8^c^
Lee et al. [30]	2 (4 %)	45 (100)	S-1 80–120 mg/day days 1–28 q6w	NA	2	8.1	4.2
	4 (9 %)	46 (100 %)	Cap 2500 mg/m^2^ days 1–14 q3w	NA	5	9.5	4.7
Lu et al. [21]	5 (11 %)	47 (100 %)	S-1 80–120 mg/day days 1–14 + Ox 130 mg/m^2^ day 1 q3w	NA	6	14.0	6.5
	4 (9 %)	47 (100 %)	S-1 80–120 mg/day days 1-14 q3w	NA	4	11.0	4
Narahara et al. [33]	5 (3 %)	155 (100 %)	S-1 80–120 mg/day days 1–21 + IRI 80 mg/m^2^ days 1–15 q5w	128 (83 %)	4	12.8	NA
	5 (3 %)	160 (100 %)	S-1 80–120 mg/day days 1–28 q6w	112 (70 %)	3	10.5	NA
Nishikawa et al. [28]	0 (0 %)	77 (100 %)	Sequential: S-1 80 mg/m^2^ days 1–28 q6w; progression PTX 50 mg/m^2^ days 1, 8, and 15 q3w; Concurrent: S-1 80 mg/m^2^ days 1-14 + PTX 50 mg/m^2^ days 1, 8, and 15 q3w	14 (18 %)	Seq: S-1 6 , PTX 4; Conc: 7.5	15.2	NA
	0 (0 %)	80 (100 %)	Sequential: 5-FU 800 mg/m^2^, days 1–5; progression PTX 80 mg/m^2^, days 1, 8, and 15 q4w Concurrent: 5-FU 600 mg/m^2^ days 1-5 + PTX 80 mg/m^2^ days 1, 8, and 15, 22 q4w	17 (21 %)	Seq: 5-FU 4, PTX 3; Conc: 6	14.2	NA
Wang et al. [35]	4 (10 %)	41 (100 %)	S-1 80–120 mg/day days 1-14 + PTX 60 mg/m^2^ days 1, 8, and 15 q4w	>50 %	6	14.0	6
	3 (7 %)	41 (100 %)	S-1 80–120 mg/day days 1–14 q4w	>50 %	5	11.0	4
Sawaki et al. [15]	3 (3 %)	68 (77 %)	S-1 80–120 mg/day days 1–28 q6w	NA	NA	8.3	3.5
	4 (4 %)	65 (73 %)	5-FU 600 mg/m^2^ bolus days 1, 8, 15, 22, 29, and 36 + Lv 250 mg/m q8w	NA	NA	10.3	4.0
Xu et al. [14]	7 (6 %)	120 (100 %)	S-1 80 mg/m^2^ days 1–21 + Cis 20 mg/m^2^ days 1–4 q5w	NA	6	10.0	5.5
	4 (3 %)	118 (100 %)	5-FU 800 mg/m^2^ days 1-5 + Cis 20 mg/m^2^ days 1-4 q4w	NA	6	10.5	4.6
Yamaguchi et al. [13]	0 (0 %)	48 (100 %)	S-1 80–120 mg/day days 1–21 + Cis 60 mg/m^2^ day 8 q5w	33 (70 %)	NA	12.6	5.6
	0 (0 %)	47 (100 %)	S-1 80–120 mg/day days 1–7 + Lv 50 mg/m^2^ days 1–7 q2w	36 (77 %)	NA	18.4	8.3
	0 (0 %)	47 (100 %)	S-1 80–120 mg/day days 1–7 + Lv 50 mg/m^2^ days 1–7 + Ox 85 mg/m^2^ day 1 q2w	35 (73 %)	NA	15.6	4.2

*Cap* capecitabine, *Cis* cisplatin, *Conc* concurrent, *DTX* docetaxel, *ECOG PS* Eastern Cooperative Oncology Group performance status, *5-FU* 5-fluorouracil, *IRI* irinotecan, *KPS* Karnofsky performance status, *Lv* leucovorin, *NA* not available, *OS* overall survival, *Ox* oxaliplatin, *PFS* progression-free survival, *PTX* paclitaxel, *q2w* every 2 weeks, *q3w* every 3 weeks, *q4w* every 4 weeks, *q5w* every 5 weeks, *q6w* every 6 weeks, *q8w* every 8 weeks, *Seq* sequential

^a^The range is given in *parentheses*

^b^The mean number of cycles was given instead of the median number of cycles received

^c^The median time to progression was given

### S-1-based therapy versus 5-FU- and capecitabine-based therapy

Eleven studies (*n* = 3135) were included in the meta-analysis: 1636 patients received S-1-based therapy, 1334 patients received 5-FU-based therapy (eight studies) and 165 patients received capecitabine-based therapy (three studies). Nine studies were conducted in Asia (*n* = 1745) and two studies were conducted in Western countries (*n* = 1372) (Table 1). We were able to extract OS and PFS data from ten and six studies. respectively, whereas ORR data were available from all 11 studies.

**Fig. 1:**
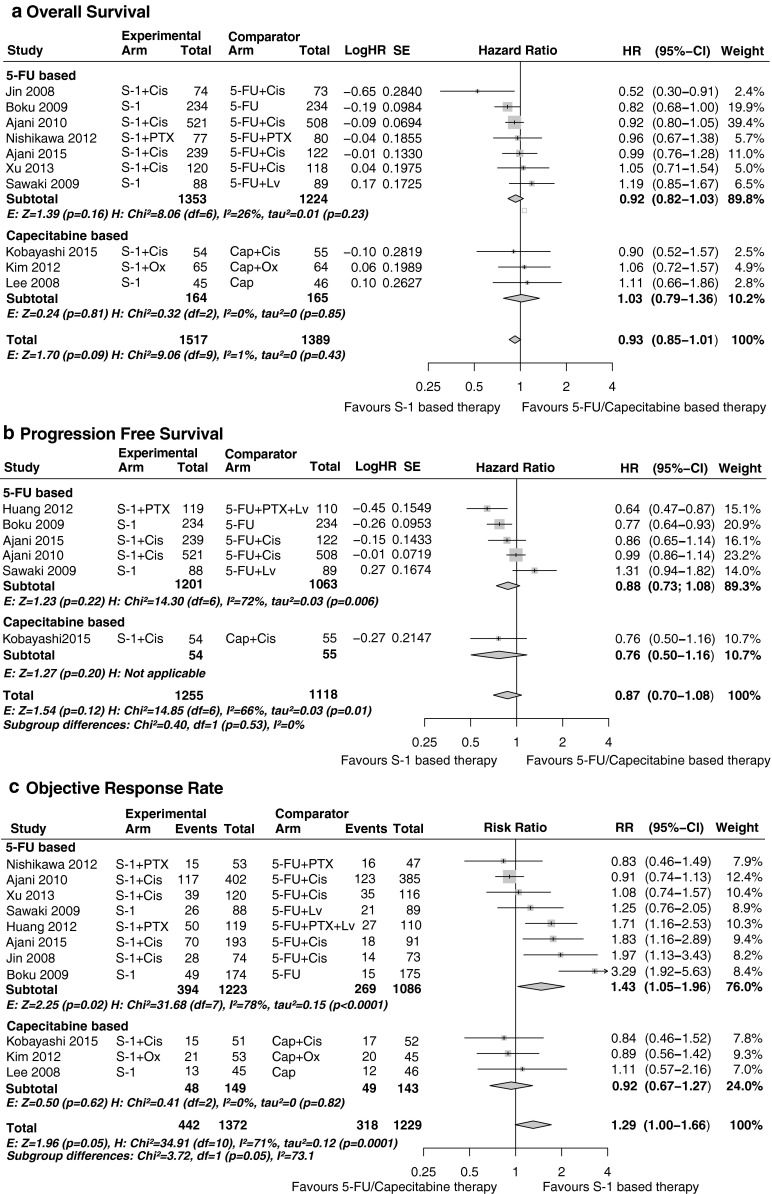
S-1-based therapy compared with 5-fluorouracil (*5-FU*)- and capecitabine (*Cap*)-based therapy: **a** overall survival; **b** progression-free survival; **c** overall response rate. *CI* confidence interval, *Cis* cisplatin, *df* degrees of freedom, *E* effect, *H* heterogeneity, *HR* hazard ratio, *Lv* leucovorin, *Ox* oxaliplatin, *PTX* paclitaxel, *RR* risk ratio, *SE* standard error

**Fig. 2:**
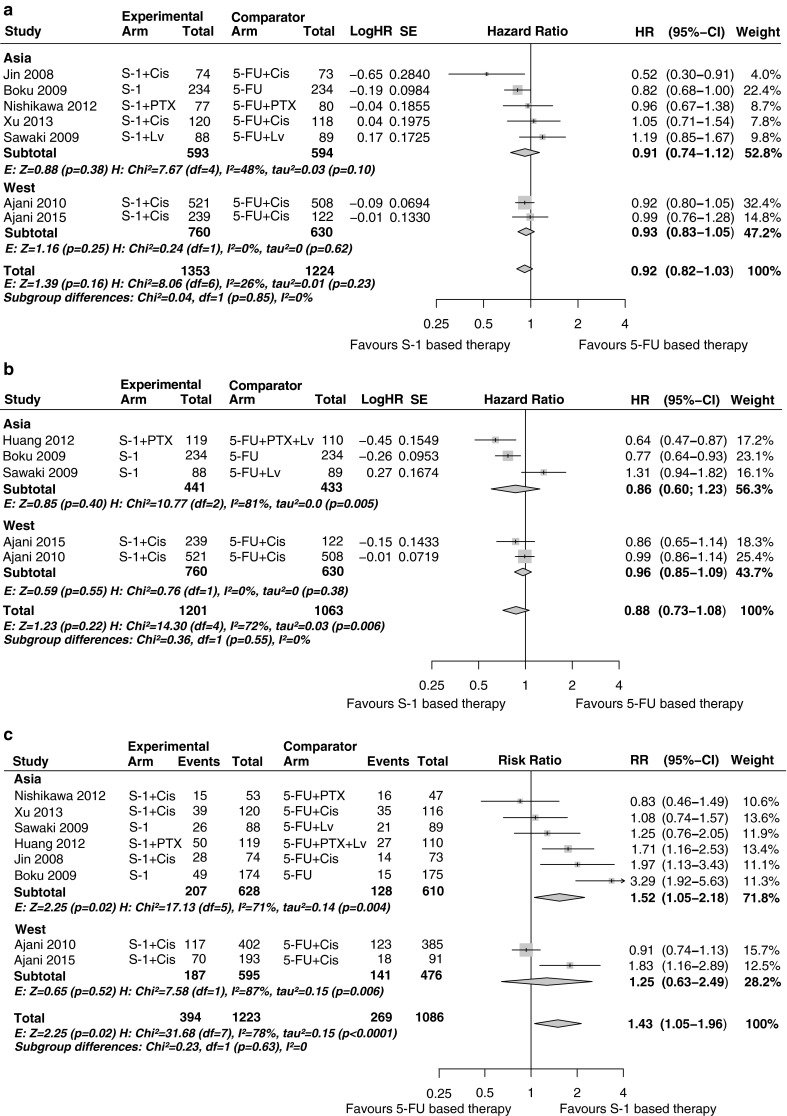
S-1-based therapy compared with 5-fluorouracil (*5-FU*)-based therapy for Asian and Western patient subgroups: **a** overall survival; **b** progression-free survival; **c** overall response rate. *Cap* capecitabine, *CI* confidence interval, *Cis* cisplatin, *df* degrees of freedom, *E* effect, *H* heterogeneity, *HR* hazard ratio, *Lv* leucovorin, *PTX* paclitaxel, *RR* risk ratio, *SE* standard error

Compared with 5-FU-based therapy, S-1-based therapy showed no difference in OS (HR 0.92, 95 % CI 0.82–1.03, *P* = 0.16) and PFS (HR 0.88, 95 % CI 0.73–1.08, *P* = 0.22), but there was a significant increase in ORR (RR 1.43, 95 % CI 1.05–1.96, *P* = 0.02) (Fig. 1). No statistically significant subgroup differences were found between Asian and Western patients in terms of OS (*P* = 0.85), PFS (*P* = 0.55) and ORR (*P* = 0.63) (Fig. 2). In the Asian population, S-1-based therapy was superior in terms of ORR compared with 5-FU-based therapy (*P* = 0.02), whereas in the Western population, statistical significance was not reached (*P* = 0.52). No significant heterogeneity was detected for OS (*I*^2^ = 26 %, *P* = 0.23); for both PFS and ORR, heterogeneity was present, with *I*^2^ = 72 % (*P* < 0.01) and *I*^2^ = 78 % (*P* < 0.001).

Compared with capecitabine-based therapy, S-1-based therapy showed no difference in OS (HR 1.03, 95 % CI 0.79–1.35, *P* = 0.81), PFS (HR 0.76, 95 % CI 0.50–1.16, *P* = 0.20) and ORR (RR 0.92, 95 % CI 0.67–1.27, *P* = 0.61) (Fig. 2). No statistically significant heterogeneity was detected.

For both comparisons, sensitivity analysis showed that the direction of the overall results was not influenced by omission of studies reported in conference abstracts only, by omission of studies stepwise according to their risk of bias, or by omission of two studies that had leucovorin in the 5-FU arm, which was the case in the studies of Sawaki et al. [15] and Huang et al. [27]. This indicates that the results are robust regarding study quality and concomitant administration of leucovorin (Table S1).

For S-1 compared with 5-FU, data were available for four haematological and 14 non-haematological grade 1–2 AEs and for five haematological and 16 non-haematological grade 3–4 AEs (Table 2). In the Western subgroup, S-1-based therapy showed significantly lower rates of febrile neutropenia, toxicity-related deaths, grade 3–4 stomatitis and mucositis and grade 1–2 diarrhoea, stomatitis and alopecia compared with 5-FU-based therapy. The rates of grade 1–2 neutropenia and hand–foot syndrome were greater with S-1 than with 5-FU.

**Table 2:** Toxicity results of S-1-based therapy compared with 5-fluorouracil (*5-FU*)-based therapy

Grade 1–2 adverse events	Western studies									Asian studies								
	S-1-based therapy		5-FU-based therapy		Estimate			Heterogeneity		S-1-based therapy		5-FU-based therapy		Estimate			Heterogeneity	
	*n*	*N*	*n*	*N*	RR^a^	*P*	Trials	*I* ^2^ (%)	*P*	*n*	*N*	*n*	*N*	RR^a^	*P*	Trials	*I* ^2^ (%)	*P*
Haematological																		
Neutropenia	143	751	69	626	1.42 (1.10–1.84)	0.008*	2	0	0.81	106	332	127	318	0.80 (0.65–0.98)	0.03*	3	0	0.88
Leucopenia	182	751	107	626	1.10 (0.92–1.32)	0.30	2	0	0.99	161	332	179	318	0.85 (0.68–1.07)	0.17	3	56	0.10
Anaemia	268	715	201	626	1.00 (0.87–1.16)	0.95	2	0	0.49	106	332	113	318	0.92 (0.76–1.11)	0.37	3	0	0.54
Thrombocytopenia	290	751	261	626	0.98 (0.57–1.71)	0.96	2	93	<0.001*	37	121	26	118	1.39 (0.90–2.14)	0.14	1	NA	NA
Non-haematological																		
Nausea	396	751	351	626	0.95 (0.86–1.05)	0.32	2	0	0.57	116	332	155	318	0.72 (0.57–0.91)	0.005*	3	33	0.22
Vomiting	281	751	262	626	1.00 (0.72–1.37)	0.98	2	66	0.09	83	332	102	318	0.75 (0.51–1.11)	0.15	3	56	0.10
Diarrhoea	167	751	193	626	0.77 (0.60–0.99)	0.05*	2	26	0.25	67	332	80	318	0.84 (0.53–1.33)	0.46	3	59	0.09
Mucositis	35	751	126	626	0.30 (0.06–1.39)	0.12	2	93	<0.001*	NA	NA	NA	NA	NA	NA	NA	NA	NA
Stomatitis	30	751	100	626	0.24 (0.12–0.48)	<0.001*	2	42	0.19	27	213	37	208	0.68 (0.27–1.68)	0.40	2	71	0.06
Anorexia	133	521	149	508	0.87 (0.71–1.06)	0.17	1	NA	NA	125	332	136	318	0.88 (0.73–1.05)	0.16	3	0	0.85
Fatigue	176	751	156	626	0.98 (0.79–1.22)	0.85	2	11	0.29	34	211	46	200	0.71 (0.32–1.62)	0.42	2	75	0.05
Asthenia	43	230	24	118	0.92 (0.59–1.44)	0.71	1	NA	NA	NA	NA	NA	NA	NA	NA	NA	NA	NA
Hand–foot syndrome	27	521	11	508	2.39 (1.20–4.27)	0.01*	1	NA	NA	NA	NA	NA	NA	NA	NA	NA	NA	NA
Neuropathy	33	751	43	626	0.56 (0.30–1.06)	0.07	2	48	0.17	3	119	1	110	2.77 (0.29–26.27)	0.37	1	NA	NA
Alopecia	31	521	103	508	0.29 (0.20–0.43)	<0.001*	1	NA	NA	21	119	13	110	1.49 (0.79–2.84)	0.22	1	NA	NA
Weight loss	165	751	149	626	0.96 (0.79–1.17)	0.72	2	0	0.36	22	92	39	90	0.55 (0.36–0.85)	0.007*	1	NA	NA
Constipation	37	230	17	118	1.12 (0.66–1.90)	0.68	1	NA	NA	34	240	42	228	0.74 (0.38–1.43)	0.37	2	55	0.14
Abdominal pain	124	751	97	626	1.14 (0.81–1.61)	0.44	2	23	0.25	17	121	7	118	2.37 (1.02–5.50)	0.04*	2	NA	NA

Grade 3–4 adverse events	Western studies									Asian studies								
	S-1-based therapy		5-FU-based therapy		Estimate			Heterogeneity		S-1-based therapy		5-FU-based therapy		Estimate			Heterogeneity	
	*n*	*N*	*n*	*N*	RR^a^	*P*	Trials	*I* ^2^ (%)	*P*	*n*	*N*	*n*	*N*	RR^a^	*P*	Trials	*I* ^2^ (%)	*P*
Haematological																		
Neutropenia	157	751	231	626	0.70 (0.30–1.63)	0.41	2	93	<0.001*	140	722	85	701	1.36 (0.66–2.77)	0.40	6	84	<0.001*
Leucopenia	57	751	80	626	0.66 (0.37–1.17)	0.16	2	53	0.15	84	722	43	701	1.56 (0.81–2.98)	0.18	6	61	0.02*
Anaemia	118	751	106	626	1.30 (0.47–3.60)	0.62	2	86	0.008*	95	722	69	701	1.42 (0.83–2.43)	0.21	6	59	0.03*
Thrombocytopenia	37	751	44	626	1.33 (0.20–8.95)	0.77	2	72	0.06	24	227	14	269	1.67 (0.38–7.36)	0.50	3	71	0.03*
Febrile neutropenia	9	521	35	508	0.25 (0.12–0.52)	<0.001*	1	NA	NA	2	353	0	342	2.87 (0.30–27.47)	0.36	2	0	0.98
Non-haematological																		
Nausea	50	751	53	626	0.83 (0.57–1.21)	0.34	2	0	0.33	25	630	32	611	0.76 (0.46–1.28)	0.30	5	0	0.98
Vomiting	52	751	54	626	0.85 (0.59–1.23)	0.39	2	0	0.57	13	488	22	469	0.59 (0.30–1.14)	0.12	5	0	0.92
Diarrhoea	28	751	25	626	1.03 (0.61–1.75)	0.91	2	0	0.74	41	722	16	701	2.03 (0.65–6.35)	0.22	6	63	0.02*
Mucositis	5	751	46	626	0.10 (0.04–0.24)	<0.001*	2	0	0.95	NA	NA	NA	NA	NA	NA	NA	NA	NA
Stomatitis	7	751	72	626	0.10 (0.05–0.20)	<0.001*	2	0	0.85	6	527	14	517	0.45 (0.18–1.13)	0.09	4	0	0.95
Anorexia	40	751	35	626	0.97 (0.63–1.51)	0.90	2	0	0.37	47	722	50	701	0.94 (0.64–1.37)	0.74	6	0	0.77
Fatigue	88	751	72	626	1.37 (0.54–3.49)	0.51	2	73	0.05	15	445	5	432	2.71 (1.02–7.21)	0.05*	3	0	0.71
Asthenia	13	230	12	118	0.53 (0.23–1.20)	0.13	1	NA	NA	NA	NA	NA	NA	NA	NA	NA	NA	NA
Hand–foot syndrome	1	521	2	508	0.49 (0.04–5.36)	0.56	1	NA	NA	3	234	0	234	7.00 (0.36–134.77)	0.20	1	NA	NA
Neuropathy	4	751	4	626	0.72 (0.17–3.18)	0.67	2	0	0.34	5	314	2	309	1.97 (0.43–9.04)	0.38	2	0	0.49
Alopecia	0	521	1	508	0.33 (0.01–7.96)	0.49	1	NA	NA	4	119	2	110	1.85 (0.35–9.89)	0.47	1	NA	NA
Weight loss	23	751	33	626	0.65 (0.39–1.09)	0.10	2	0	0.81	0	80	2	77	0.19 (0.01–3.95)	0.29	1	NA	NA
Constipation	2	230	0	118	2.58 (0.12–53.22)	0.54	1	NA	NA	0	121	1	118	0.33 (0.01–7.90)	0.49	1	NA	NA
Abdominal pain	51	751	29	626	1.62 (0.82–3.20)	0.17	2	22	0.26	2	121	0	118	4.88 (0.24–100.52)	0.30	1	NA	NA
Serious adverse events	170	751	182	626	0.83 (0.55–1.23)	0.35	2	72	0.06	4	119	2	110	1.85 (0.35–9.89)	0.47	1	NA	NA
Toxicity-related death	14	571	26	626	0.51 (0.27–0.96)	0.04*	2	0	0.99	2	353	1	342	1.52 (0.19–12.30)	0.69	2	0	0.59

A risk ratio (*RR*) greater than 1 represents a beneficial effect of S-1-based therapy

*NA* not available

* P < 0.05

^a^The 95 % confidence interval is given in *parentheses*

In the Asian subgroup, S-1-based therapy showed a significantly increased incidence of grade 3–4 fatigue and grade 1–2 abdominal pain but a lower incidence of grade 1–2 neutropenia, nausea and weight loss compared with 5-FU-based therapy. The incidence of febrile neutropenia, serious AEs or toxicity-related deaths was not different between both arms.

For S-1 compared with capecitabine, data were available for four haematological and 13 non-haematological grade 1–2 AEs and for five haematological and 12 non-haematological grade 3–4 AEs (Table 3). Lower rates of grade 3–4 neutropenia and grade 1–2 hand–foot syndrome were found with S-1-based therapy compared with capecitabine-based therapy. The incidence of febrile neutropenia, serious AEs or toxicity-related deaths was not different between both arms.

**Table 3:** Toxicity results of S-1-based therapy compared with capecitabine-based therapy

	Grade 1–2									Grade 3–4								
	S-1-based therapy		Capecitabine-based therapy		Estimate			Heterogeneity		S-1-based therapy		Capecitabine-based therapy		Estimate			Heterogeneity	
	*n*	*N*	*n*	*N*	RR^a^	*P*	Trials	*I* (%)	*P*	*n*	*N*	*n*	*N*	RR^a^	*P*	Trials	*I* (%)	*P*
Haematological																		
Neutropenia	59	163	65	163	0.91 (0.69–1.19)	0.50	3	0	0.39	13	163	25	163	0.52 (0.27–0.97)	0.04*	3	0	0.93
Leucopenia	42	107	41	108	1.05 (0.65–1.71)	0.83	2	39	0.20	6	107	3	108	1.98 (0.50–7.90)	0.33	2	0	0.60
Anaemia	126	163	132	163	0.94 (0.84–1.05)	0.29	3	0	0.38	23	163	18	163	1.31 (0.57–3.01)	0.52	3	42	0.18
Thrombcytopenia	68	163	77	163	0.87 (0.69–1.10)	0.24	3	0	0.53	11	163	11	163	1.02 (0.47–2.20)	0.97	3	0	0.77
Febrile neutropenia	NA	NA	NA	NA	NA	NA	NA	NA	NA	3	163	3	163	1.03 (0.22–4.93)	0.97	3	0	0.69
Non-haematological																		
Nausea	83	163	71	163	1.19 (0.90–1.58)	0.23	2	36	0.21	8	163	9	163	0.8 (0.31–2.07)	0.65	3	0	0.43
Vomiting	46	163	46	163	0.98 (0.61–1.56)	0.92	3	40	0.19	4	163	4	163	0.97 (0.25–3.75)	0.96	3	0	0.66
Diarrhoea	45	163	45	163	0.99 (0.70–1.41)	0.98	3	0	0.61	9	163	4	163	1.55 (0.24–10.16)	0.65	3	44	0.17
Mucositis	NA	NA	NA	NA	NA	NA	NA	NA	NA	2	65	0	64	4.92 (0.24–100.60)	0.30	1	NA	NA
Stomatitis	23	98	36	99	0.67 (0.23–1.90)	0.45	2	80	0.02*	0	56	2	55	0.20 (0.01–4.00)	0.29	1	NA	NA
Anorexia	88	163	97	163	0.89 (0.74–1.07)	0.21	3	0	0.50	16	163	12	163	1.34 (0.66–2.71)	0.42	3	0	0.95
Fatigue	22	56	23	55	0.94 (0.60–1.47)	0.79	1	NA	NA	3	56	3	55	0.98 (0.21–4.66)	0.98	1	NA	NA
Asthenia	57	107	57	108	1.04 (0.83–1.30)	0.73	2	0	0.58	4	107	9	110	0.50 (0.14–1.78)	0.29	2	10	0.29
Hand–foot syndrome	14	163	59	163	0.25 (0.15–0.43)	<0.001*	3	0	0.52	0	163	5	163	0.25 (0.04–1.46)	0.12	3	0	0.92
Neuropathy	28	121	39	119	0.56 (0.17–1.80)	0.33	2	71	0.06	2	65	3	64	0.66 (0.11–3.80)	0.64	1	NA	NA
Alopecia	0	56	4	55	0.11 (0.01–1.98)	0.13	1	NA	NA	NA	NA	NA	NA	NA	NA	NA	NA	NA
Weight loss	13	56	11	55	1.16 (0.57–2.36)	0.68	1	NA	NA	1	56	1	55	0.98 (0.06–15.31)	0.99	1	NA	NA
Constipation	0	56	1	55	0.33 (0.01–7.87)	0.49	1	NA	NA	NA	NA	NA	NA	NA	NA	NA	NA	NA
Abdominal pain	27	98	24	99	1.16 (0.62–2.16)	0.64	2	35	0.22	5	98	1	99	3.75 (0.63–22.27)	0.15	2	0	0.86

A risk ratio (*RR*) greater than 1 represents a beneficial effect S-1-based therapy

*NA* not available

* *P* < 0.05

^a^The 95 % confidence interval is given in *parentheses*

### S-1-based combination therapy versus S-1 monotherapy

For this comparison, eight studies (*n* = 1808) were included in the meta-analysis, with 927 and 881 patients in the S-1 combination therapy group and the S-1 monotherapy group, respectively. Four different combination therapies were compared with S-1 monotherapy: S-1 plus cisplatin therapy (*n* = 544 patients, three studies), S-1 plus oxaliplatin therapy (*n* = 190, two studies), S-1 plus taxane therapy (*n* = 717, two studies) and S-1 plus irinotecan therapy (*n* = 404, two studies). All studies were conducted in Asia: three studies in China, four studies in Japan, and one study in both Japan and Korea (Table 1). We extracted the HRs and 95 % CIs from seven studies for OS and from five studies for PFS. ORRs were available from all eight studies.

**Fig. 3:**
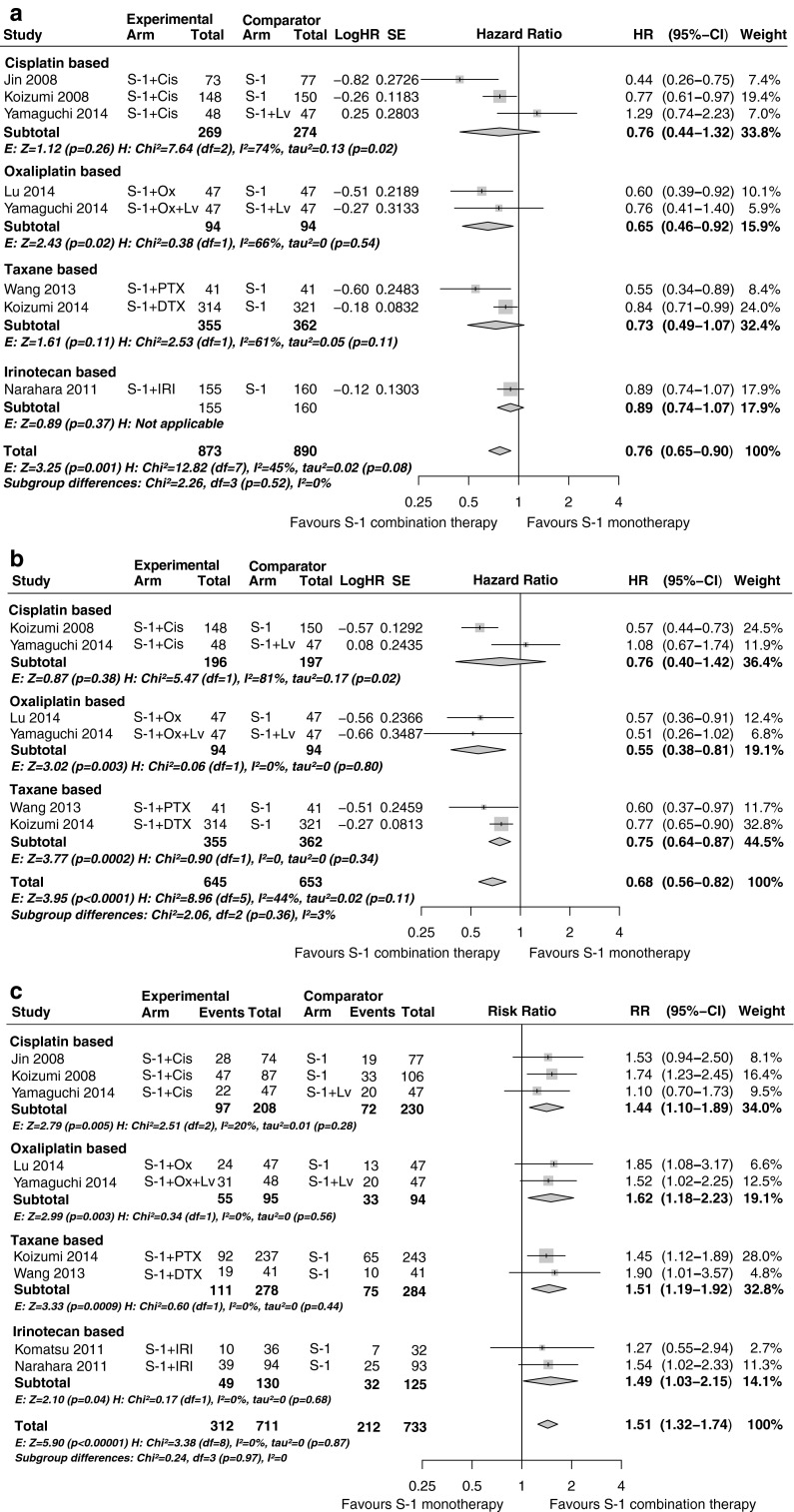
S-1-based combination therapy compared with S-1 monotherapy: **a** overall survival; **b** progression-free survival; **c** overall response rate. *CI* confidence interval, *Cis* cisplatin, *df* degrees of freedom, *DTX* docetaxel, *E* effect, *H* heterogeneity, *HR* hazard ratio, *IRI* irinotecan, *Lv* leucovorin, *NA* not available, *Ox* oxaliplatin, *PTX* paclitaxel, *RR* risk ratio, *SE* standard error

The pooled estimates of S-1 combination therapy versus S-1 monotherapy were superior for OS (HR 0.76, 95 % CI 0.65–0.89, *P* < 0.001), PFS (HR 0.68, 95 % CI 0.56–0.82, *P* < 0.001) and ORR (RR 1.51, 95 % CI 1.32–1.74, *P* < 0.001) (Fig. 3). Subgroup analyses showed that ORR was significantly better for all four combination therapies and showed no evidence of heterogeneity (*I*^2^ = 0 %, *P* = 0.95). However, only S-1 plus oxaliplatin therapy showed significant estimates for both OS and PFS compared with S-1 monotherapy, whereas OS was not significant for S-1 combined with irinotecan, cisplatin or a taxane. PFS was statistically significant for S-1 plus taxane therapy, but not for S-1 plus cisplatin therapy or S-1 plus irinotecan therapy.

Heterogeneity was explored in subanalyses and sensitivity analyses (Table S2). For the cisplatin-based and taxane-based subgroup analyses, the non-significant effect might by due to some heterogeneity among the studies (OS *I*^2^ = 45.0 %, *P* = 0.08; PFS *I*^2^ = 44 %, *P* = 0.11). When studies were stratified according to region, a significant subgroup difference between Chinese studies and Japanese studies was found in OS (*P* < 0.005). No subgroup differences for region were found in PFS (*P* = 0.38) and ORR (*P* = 0.88). Furthermore, no significant fluctuations in the overall results were detected with sensitivity analysis according to study quality and concomitant administration of leucovorin, which was the case with the comparison of S-1 plus cisplatin therapy with S-1 plus leucovorin therapy in the study of Yamaguchi et al. [13].

Data were available for four haematological and 12 non-haematological grade 1–2 AEs and for five haematological and 11 non-haematological grade 3–4 AEs. Compared with S-1 monotherapy, S-1-based doublets were associated with an increased rate of grade 3–4 neutropenia, leucopenia and stomatitis and with an increased rate of grade 1–2 leucopenia, anaemia, thrombocytopenia, lymphocytopenia, anorexia, fatigue and alopecia (Table S3).

**Fig. 4:**
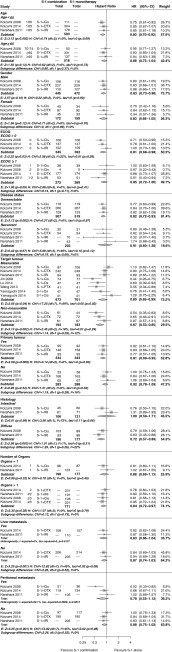
Stratified overall survival (OS) results for S-1 combination therapy versus S-1 monotherapy. Forest plot of OS results for S-1-based combination therapy versus S-1 monotherapy stratified per patient subgroup. For target tumour more than three studies are shown because these studies included only patients with measurable lesions. Pooled sample sizes are stated for S-1 combination therapy and S-1 monotherapy groups if separate sample sizes were not available in the study report.  *CI* confidence interval, *Cis* cisplatin, *df* degrees of freedom, *DTX* docetaxel, *E* effect, *ECOG* Eastern Cooperative Oncology Group performance status, *H* heterogeneity, *HR* hazard ratio, *IRI* irinotecan, *Ox* oxaliplatin, *PTX* paclitaxel

To identify subgroups that may benefit most from S-1 combination therapy compared with S-1 monotherapy, three large phase III Japanese studies (*n* = 1248) reporting a stratified analysis for OS could be used (Fig. 4) [32, 33]. The pooled effect size for these three studies was HR 0.82 (95 % CI 0.72–0.93). A trend toward significant subgroup differences in favour of S-1 combination therapy was found in favour of patients with diffuse-type histological features compared with patients with intestinal-type histological features (*P* = 0.06; HR < 0.80) and patients with measurable disease compared with patients with non-measurable disease (*P* = 0.06; HR < 0.80). Furthermore, subgroups with peritoneal metastases showed a non-significant but clinically relevant HR (0.80 or less) in favour of S-1 combination therapy. No other potential predictive factors were identified.

### Publication bias

Funnel plots did not show significant asymmetry and Egger’s test was not significant for S-1-based therapy versus 5-FU/capecitabine-based therapy in terms of OS (*P* = 0.75), PFS (*P* = 0.82), and ORR (*P* = 0.73) and for S-1-based combination therapy versus S-1 monotherapy in terms of OS (*P* = 0.08), PFS (*P* = 0.71) and ORR (*P* = 0.96) (Figure S3).

## Discussion

Previous meta-analyses have suggested that 5-FU may be replaced by S-1 in first-line therapy for advanced gastric cancer because of a survival benefit in favour of S-1 [3, 4]. Our updated meta-analysis does not confirm this finding. Although a higher ORR was observed for S-1-based therapy versus 5-FU-based therapy, OS and PFS were not significantly prolonged. The pooled OS and PFS effect sizes of the two recently conducted Western studies, the FLAGS and DIGEST trials, were comparable to the pooled OS and PFS effect sizes of all Asian studies. This suggests that S-1 may have similar efficacy in both Western and Asian patients. However, in Western patients S-1-based therapy did have clear clinically relevant advantages in terms of the toxicity profile over 5-FU-based therapy—namely, lower rates of febrile neutropenia, toxicity-related-deaths and grade 3–4 mucositis and stomatitis, whereas the toxicity profiles of S-1 and 5-FU in Asian patients showed no clinically relevant differences, except a higher rate of grade 3–4 fatigue and lower rates of grade 1–2 neutropenia and nausea. This indicates that S-1 is well tolerated in Western patients with its current dosing as used in the FLAGS and DIGEST trials.

Also, S-1 was not more effective than capecitabine in Asian patients. In the West, it has been suggested that capecitabine may be replaced by S-1 in the case of hand–foot syndrome. This meta-analysis shows that the incidence of grade 1–2 hand–foot syndrome was significantly lower with S-1 than with capecitabine. We stress that hand–foot syndrome specifically can have a severe impact on quality of life, because capacitabine is usually given for a longer time. Moreover, in a previous review which also included studies in metastatic colorectal cancer, a significantly lower rate of grade 3–4 hand–foot syndrome was observed for S-1 (0.3 %) compared with capecitabine (3.1 %); *P* < 0.001 [7]. Also, in our meta-analysis there were fewer observations of grade 3–4 hand–foot syndrome with S-1 (0.0 %) versus capecitabine (3.1 %), but the numbers were too low to reach statistical significance. Because all capecitabine studies were conducted in Asia, we should interpret our findings with caution for Western populations..

This is the first meta-analysis to examine the differential efficacy of combination therapy and monotherapy in patients with different baseline factors and can aid in clinical decision making. Overall, we showed that S-1 combination therapy is more efficacious than S-1 monotherapy. Importantly, our meta-analysis of stratified data from the three largest studies suggests that patients with disease characteristics associated with poor prognosis, such as non-measurable lesion, diffuse-type histological features and peritoneal metastasis, may have increased benefit from combination therapy.

The pooled result for the OS benefit of taxane combinations was not convincing because of heterogeneity. However, the HR (0.73) may be considered clinically meaningful and the PFS was significantly prolonged. Improvement of PFS may also be an important finding, because PFS is less prone to the influence of second-line therapy than OS. More grade 1–2 and grade 3–4 haematological toxicity as well as gastrointestinal toxicity occurred with combination therapy compared with monotherapy, which was in line with other combination chemotherapy regimens including a fluoropyrimidine combined with platinum compounds [36, 37], taxanes [38, 39] or irinotecan [37, 40].

Our study has some limitations. First, we did not take specific dosing regimens into account, which could have impacted our results. With pooled data analyses, including meta-analysis, it is often not possible to investigate to what extent dose differences may have influenced the results of the meta-analysis. Also, in some studies, leucovorin was added to fluoropyrimidine therapy. Leucovorin increases the intracellular pool of 5,10-methylenetetrahydrofolate, thereby enhancing thymidylate synthase inhibition by fluorodeoxyuridine monophosphate [41]. This mechanism of action implies that leucovorin should be regarded not as an additional cytotoxic agent but rather as a modulator of fluoropyrimidine efficacy and toxicity. We conducted sensitivity analyses in which we omitted the studies in which leucovorin was concomitantly administrated with one of the S-1 or 5-FU regimens. This did not affect the pooled effect sizes of all comparisons. Furthermore, most of the fluoropyrimidine dosing regimens of the studies included in our review were similar. Especially the dosing of S-1 is fairly constant among different studies.

A second limitation is that the heterogeneity due to the difference in OS effect size in the Chinese subgroup and Japanese subgroup may somewhat complicate the interpretation of the S-1 combination therapy versus S-1 monotherapy analysis. Two of the Chinese studies were single-centre studies, whereas all Japanese studies were multicentre studies and therefore may have higher quality. However, the sensitivity analysis according to the risk of bias did not suggest major fluctuations in results. Whether there is a real difference in efficacy for combination therapy between Chinese and Japanese populations or whether this is purely a methodological issue remains unclear and should be addressed in larger and more qualitatively sound studies with Chinese patients.

In summary, S-1-based therapy showed no difference in survival compared with 5-FU- and capecitabine-based therapy but has a higher ORR compared with 5-FU-based therapy. In terms of clinical relevance, the toxicity profile of S-1 compared with 5-FU was clearly more advantageous in Western patients. Also, S-1 showed a better toxicity profile compared with capecitabine, with a lower incidence of hand–foot syndrome. In general, S-1 combination therapy is superior to S-1 monotherapy in terms of efficacy, and patients with poor prognosis disease characteristics may benefit most from S-1 combination therapy, although S-1 combinations were more toxic than S-1 alone. Our findings suggest that S-1-based regimens are effective and tolerable as first-line treatment of advanced gastric cancer in both Asian and Western countries.

## Electronic supplementary material

Below is the link to the electronic supplementary material.

Supplementary material 1 (DOCX 22 kb) Document S1. Full literature search strategy

Supplementary material 2 (DOCX 21 kb) Figure S1. Flowchart of included studies. Left path: database screening. Right path: conference reports and ClinicalTrials.gov screening

Supplementary material 3 (PDF 578 kb) Figure S2. Risk of bias assessment. A: Overall survival. B: Progression-free-survival and objective response rate. For Jin 2008 the conference presentation indicated low risk of bias on most items. Since the primary outcome OS would not be influenced by absence of blinded imaging review, this item was not scored as unknown or high of bias for overall survival. Abbreviations: +: low risk of bias, ?: unknown risk of bias, −: high risk of bias

Supplementary material 4 (PDF 395 kb) Figure S3. Funnels plots for assessment of publication bias. OS, PFS and ORR for S-1-based therapy versus 5-FU- and capecitabine-based therapy (A-C) and for S-1-based combination therapy versus S-1 monotherapy (D-F)

Supplementary material 5 (DOCX 16 kb) Table S1. Sensitivity analysis of S-1-based therapy compared with 5-FU- and capecitabine-based therapy. Left: S-1-based therapy versus 5-FU-based therapy. Right: S-1-based therapy versus capecitabine-based therapy. Exploring heterogeneity by sensitivity analysis of omitting studies according to their risk of bias. *CI* confidence interval, *NA* not available, *RR* risk ratio

Supplementary material 6 (DOCX 15 kb) Table S2. Sensitivity analyses for S-1 combination therapy compared with S-1 monotherapy. Exploring heterogeneity by sensitivity analysis of omitting studies according to their risk of bias and subanalysis of study regions within Asia. The 95 % confidence intervals of China and Japan do not overlap and the point estimate (hazard ratio) of China was 0.30 lower compared with that of Japan. This may be a possible explanation for the heterogeneity in the main analysis. *CI* confidence interval, *NA* not available, *RR* risk ratio

Supplementary material 7 (DOCX 22 kb) Table S3. Toxicity results of S-1-based combination therapy compared with S-1 monotherapy. A risk ratio (*RR*) greater than 1 represents a beneficial effect for the experimental arm. *CI* confidence interval, *N* safety sample size, *NA* not available
