# Supplementary material for: The efficacy and safety of S-1-based regimens in the first-line treatment of advanced gastric cancer: a systematic review and meta-analysis
Source: Gastric Cancer. 2016 Jan 11;19:696–712. doi: 10.1007/s10120-015-0587-8 (PMC4906062; doi:10.1007/s10120-015-0587-8)
Supplement: Supplementary file 1 — Supplementary material 1 (DOCX 22 kb) Document S1. Full literature search strategy [file 10120_2015_587_MOESM1_ESM.docx]

**Supplementary Document S1. Full search strategy**

**Medline (Pubmed)**

"Stomach Neoplasms"[Mesh] AND "S 1 (combination)"[Supplementary Concept] AND Clinical Trial[ptyp]

Results: 228

**The Cochrane Library (CENTRAL)**

1. MeSH descriptor: [Stomach Neoplasms] explode all trees

2. S-1

3. #1 and #2

**EMBASE (via Ovid)**

|  | 1 | exp stomach cancer/ |
| --- | --- | --- |
|  | 2 | limit 1 to human |
|  | 3 | exp gimeracil plus oteracil potassium plus tegafur/ |
|  | 4 | 1 and 2 and 3 |
|  | 5 | clinical trial/ |
|  | 6 | 4 and 5 |
|  | 7 | exp "randomized controlled trial (topic)"/ or exp controlled clinical trial/ or exp "phase 2 clinical trial (topic)"/ or exp "clinical trial (topic)"/ or trial.mp. or exp "controlled clinical trial (topic)"/ or exp "phase 3 clinical trial (topic)"/ |
| **** | **8** | **4 and 7** |

**www.clinicaltrials.gov**

S-1(search terms) | "Stomach Neoplasms" (condition)| Phase 2, 3 (filters)

**American Society of Clinical Oncology (ASCO)**

<http://www.ascopubs.org/search>

Abstract and title: ‘S-1’ AND ‘gastric’ (all words).

**European Society for Medical Oncology (ESMO)**

<http://annonc.oxfordjournals.org/search>

Abstract and title: ‘S-1’ AND ‘gastric’ (all words).
