# Supplementary material for: The efficacy and safety of S-1-based regimens in the first-line treatment of advanced gastric cancer: a systematic review and meta-analysis
Source: Gastric Cancer. 2016 Jan 11;19:696–712. doi: 10.1007/s10120-015-0587-8 (PMC4906062; doi:10.1007/s10120-015-0587-8)
Supplement: Supplementary file 2 — Supplementary material 2 (DOCX 21 kb) Figure S1. Flowchart of included studies. Left path: database screening. Right path: conference reports and ClinicalTrials.gov screening [file 10120_2015_587_MOESM2_ESM.docx]

**Supplementary Figure S1. Flowchart of included studies**

References derived from clinicaltrials.gov (n=87), ASCO (n=278) and ESMO (n=45) until May 2015

References derived from Medline (n=228), EMBASE (n=245) and CENTRAL (n=39) until May 2015 (total n=501)

Excluded based on title and abstract: clinicaltrials.gov (n=84), ASCO (n=269) and ESMO (n=42)

Removed duplicates (n=147)

Unique references for screening based on title and abstract (n=354)

Relevant references: clinicaltrials.gov (n=5), ASCO (n=9), ESMO (n=3)

Excluded based on title and abstract (n=326)

**Excluded after detailed assessment:**

Clinicaltrials.gov: n=3

Already published as full articles: Koizumi 2014, Ajani 2010 and Lee 2008.

ASCO n=5

Meeting abstracts from already published studies

- Koizumi 2014 (n=2)

- Narahara 2011 (n=2)

- Koizumi 2008 (n=1)

ESMO: n=0

**References for full-text assessment (n=28)**

S-1 based vs non-S-1 based (n=20)

S-1 combination vs S-1 alone: (n=8)

**Excluded after detailed assessment (n=15)**

S-1 based vs 5-FU/Capecitabine based (n=13):

- Outdated preliminary results (n=4)

- Post-hoc analyses (n=3)

- Cohort or retrospective studies (n=5)

- No full text and no description of the design and data in abstract (n=1)

S-1 combination vs S-1 alone (n=2):

- Outdated preliminary result (n=1)

- Pharmacokinetic study (n=1)

**Additional unique studies (n=6):**

Kobayashi 2015, Yamaguchi 2014, Ajani 2015, Jin 2008, Sawaki 2009 and Xu 2013

clinicaltrials.gov (n=2): Kobayashi 2015 and Jin 2008.

ASCO (n=4): Jin 2008, Kobayashi 2015, Ajani 2015 and Xu 2013

ESMO (n=2): Yamaguchi 2014 and Sawaki 2009

**Studies eligible for meta-analysis (n=18)**

**S-1 based versus 5-FU based therapy:** n=**8**

- Database (n=4): Ajani 2010, Huang 2013, Nishikawa 2012, Boku 2009.

- Conferences (n=4): Jin 2008, Ajani 2015, Sawaki 2009, Xu 2013.

**S-1 based versus capecitabine based therapy: n=3**

- Database (n=2): Kim 2012, Lee 2008

- Conferences (n=1): Kobayashi 2015

**S-1-based combination therapy versus S-1 monotherapy: n=8**

- Database (n=6): Koizumi 2008, Koizumi 2014, Komatsu 2011, Lu 2014, Narahara 2011 and Wang 2013

- Conference (n=2): Jin 2008 and Yamaguchi 2014

**Stratified analysis for S-1 based combination therapy versus S-1 monotherapy: n=3**

- Database (n=3): Koizumi 2008, Koizumi 2014 and Narahara 2011

(Jin 2008 was eligible for more comparisons of this review).
