# Supplementary material for: The efficacy and safety of S-1-based regimens in the first-line treatment of advanced gastric cancer: a systematic review and meta-analysis
Source: Gastric Cancer. 2016 Jan 11;19:696–712. doi: 10.1007/s10120-015-0587-8 (PMC4906062; doi:10.1007/s10120-015-0587-8)
Supplement: Supplementary file 3 — Supplementary material 3 (PDF 578 kb) Figure S2. Risk of bias assessment. A: Overall survival. B: Progression-free-survival and objective response rate. For Jin 2008 the conference presentation indicated low risk of bias on most items. Since the primary outcome OS would not be influenced by absence of blinded imaging review, this item was not scored as unknown or high of bias for overall survival. Abbreviations: +: low risk of bias, ?: unknown risk of bias, −: high risk of bias [file 10120_2015_587_MOESM3_ESM.pdf]

Supplementary Figure S2. Risk of bias assessment.

A Overall Survival

	Random sequence generation (selection bias)	Allocation concealment (selection bias)	Blinding of outcome assessment (detection bias)	Incomplete outcome data (attrition bias)	Selective reporting (reporting bias)	Other bias
Ajani 2013	?	?	+	+	+	+
Ajani 2015	?	?	+	+	+	?
Boku 2009	+	+	+	+	+	+
Huang 2012	?	?	+	+	+	+
Jin 2008	+	+	+	+	+	?
Kim 2012	+	+	+	+	+	+
Kobayashi 2015	?	?	+	+	+	?
Koizumi 2008	+	+	+	+	+	+
Koizumi 2014	?	?	+	+	+	+
Komatsu 2011	+	+	+	+	+	+
Lee 2008	+	+	+	+	+	+
Lu 2014	+	+	+	+	+	?
Narahara 2011	+	+	+	+	+	+
Nishikawa 2012	+	+	+	+	+	+
Sawaki 2009	?	?	+	+	+	?
Wang 2013	+	+	+	+	+	?
Xu 2013	?	?	+	+	+	?
Yamaguchi 2014	?	?	+	+	+	?

B Progression Free Survival and Objective Response Rate

	Random sequence generation (selection bias)	Allocation concealment (selection bias)	Blinding of outcome assessment (detection bias)	Incomplete outcome data (attrition bias)	Selective reporting (reporting bias)	Other bias
Ajani 2013	?	?	+	+	+	+
Ajani 2015	?	?	?	+	+	?
Boku 2009	+	+	+	+	+	+
Huang 2012	?	?	?	+	+	+
Jin 2008	+	+	+	+	+	?
Kim 2012	+	+	?	+	+	+
Kobayashi 2015	?	?	+	+	+	?
Koizumi 2008	+	+	+	+	+	+
Koizumi 2014	?	?	+	+	+	+
Komatsu 2011	+	+	+	+	+	+
Lee 2008	+	+	+	+	+	+
Lu 2014	+	+	?	+	+	?
Narahara 2011	+	+	+	+	+	+
Nishikawa 2012	+	+	?	+	+	+
Sawaki 2009	?	?	?	+	+	?
Wang 2013	+	+	?	+	+	?
Xu 2013	?	?	+	+	+	?
Yamaguchi 2014	?	?	?	+	+	?
