# Supplementary material for: The efficacy and safety of S-1-based regimens in the first-line treatment of advanced gastric cancer: a systematic review and meta-analysis
Source: Gastric Cancer. 2016 Jan 11;19:696–712. doi: 10.1007/s10120-015-0587-8 (PMC4906062; doi:10.1007/s10120-015-0587-8)

Supplementary Figure S3. Funnelplots for assessment of publication bias.

S-1 based therapy versus 5-FU/Capecitabine based therapy

A Overall Survival

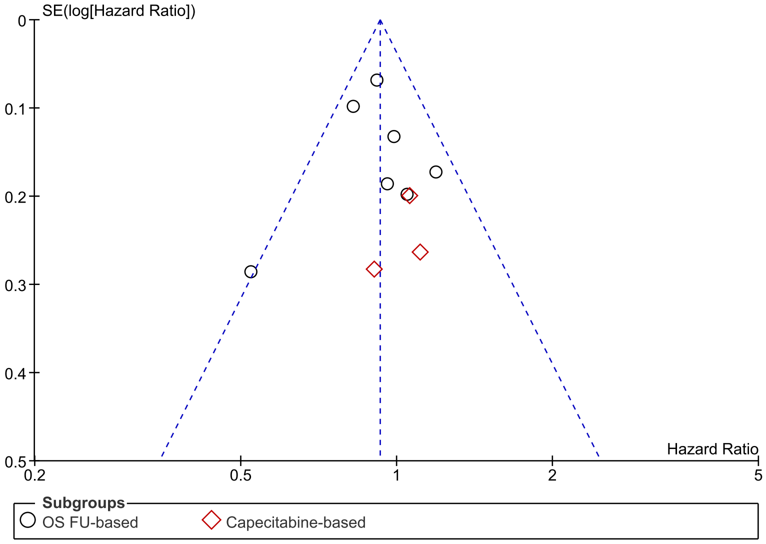

B Progression Free Survival

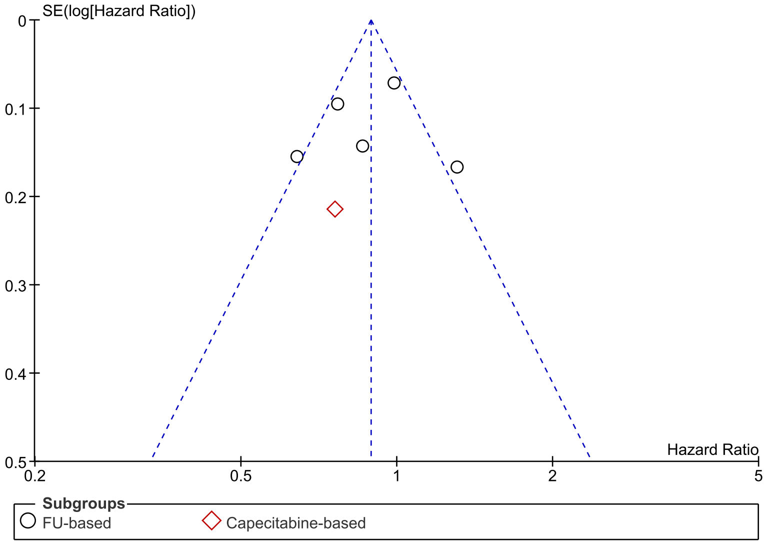

C Objective Response Rate

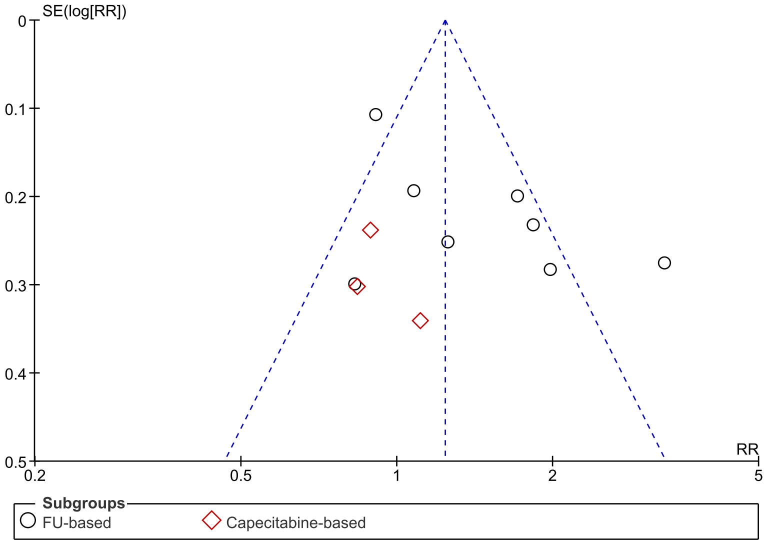

S-1 combination therapy versus S-1 monotherapy

D Overall Survival

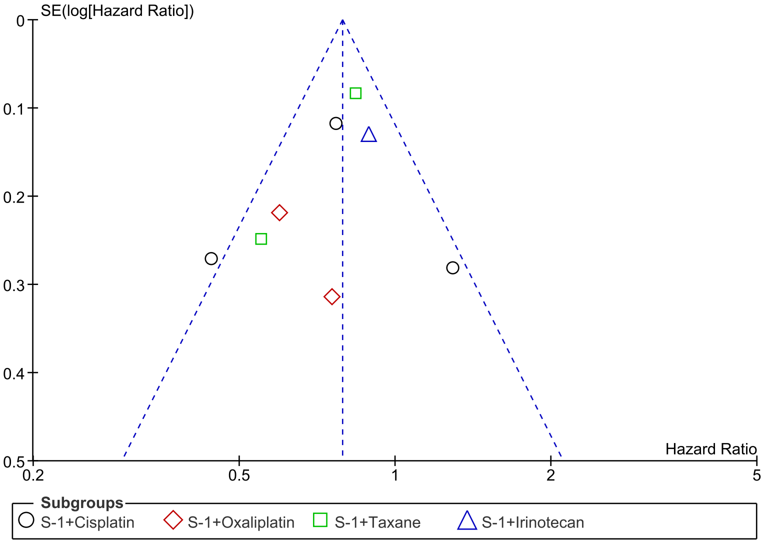

E Progression Free Survival

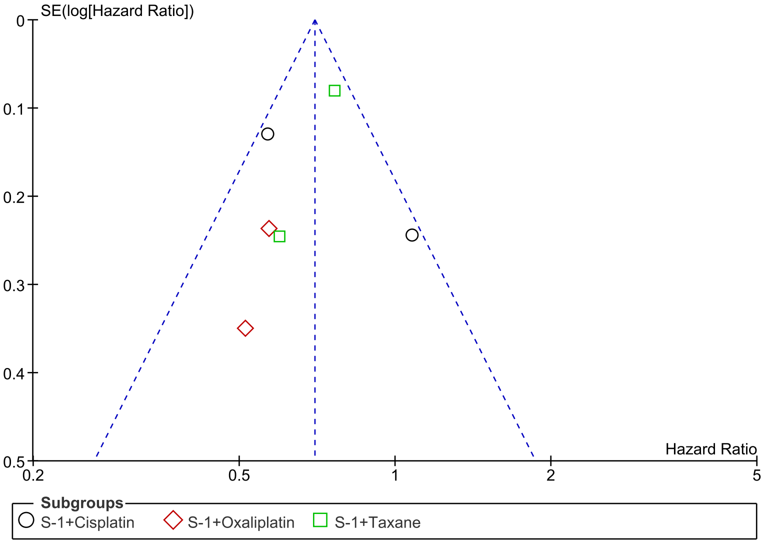

F Objective Response Rate

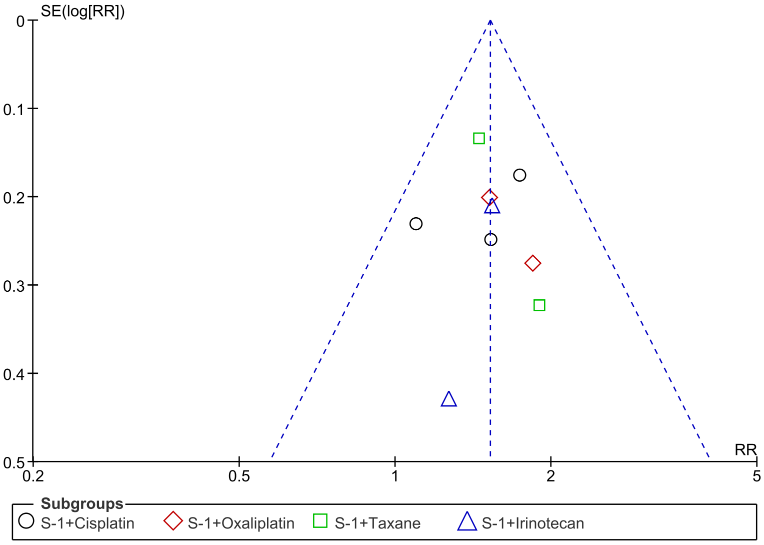

Supplement: Supplementary file 4 — Supplementary material 4 (PDF 395 kb) Figure S3. Funnels plots for assessment of publication bias. OS, PFS and ORR for S-1-based therapy versus 5-FU- and capecitabine-based therapy (A-C) and for S-1-based combination therapy versus S-1 monotherapy (D-F) [file 10120_2015_587_MOESM4_ESM.pdf]
