# Supplementary material for: The efficacy and safety of S-1-based regimens in the first-line treatment of advanced gastric cancer: a systematic review and meta-analysis
Source: Gastric Cancer. 2016 Jan 11;19:696–712. doi: 10.1007/s10120-015-0587-8 (PMC4906062; doi:10.1007/s10120-015-0587-8)
Supplement: Supplementary file 5 — Supplementary material 5 (DOCX 16 kb) Table S1. Sensitivity analysis of S-1-based therapy compared with 5-FU- and capecitabine-based therapy. Left: S-1-based therapy versus 5-FU-based therapy. Right: S-1-based therapy versus capecitabine-based therapy. Exploring heterogeneity by sensitivity analysis of omitting studies according to their risk of bias. CI confidence interval, NA not available, RR risk ratio [file 10120_2015_587_MOESM5_ESM.docx]

**Supplementary Table S1.** **Sensitivity analysis of S-1 based therapy compared to 5-FU and Capecitabine based therapy**

|  | **S-1 based versus 5-FU based therapy** | | |
| --- | --- | --- | --- |
|  | **OS HR (95%CI)** | **PFS HR (95%CI)** | **ORR RR (95%CI)** |
| **Full analysis set** | 0.92 (0.82‒1.03) | 0.88 (0.73‒1.08) | 1.43 (1.05‒1.96) |
| **Sensitivity analysis by risk of bias items** |  |  |  |
| Studies with one or more unknown risk items omitted | 0.85 (0.72‒1.01) | 0.77 (0.64‒0.93) | 3.29 (1.92‒5.63) |
| Studies with two or more unknown risk of bias items omitted | 0.80 (0.63‒1.03) | 0.77 (0.64‒0.93) | 1.76 (0.80‒3.87) |
| Studies with three or more unknown risk of bias items omitted | 0.86 (0.75‒0.99) | 0.88 (0.69‒1.12) | 1.46 (0.77‒2.77) |
| Conference abstracts omitted | 0.89 (0.80‒0.99) | 0.81 (0.64‒1.03) | 1.51 (0.92‒2.48) |
| Low risk studies omitted | 0.95 (0.83‒1.09) | 0.92 (0.72‒1.17) | 1.28 (0.98‒1.67) |
| **Studies containing 5-FU + Leucovorin omitted*** | 0.90 (0.81‒1.00) | 0.87 (0.77‒1.04) | 1.43 (0.95‒2.15) |

Notes: * Sawaki 2009 and Huang 2013 were omitted in this sensitivity analysis

|  | **S-1 based versus capecitabine based therapy** | | |
| --- | --- | --- | --- |
|  | **OS HR (95%CI)** | **PFS HR (95%CI)** | **ORR RR (95%CI)** |
|  |  |  |  |
| **Full analysis set** | 1.03 (0.79‒1.35) | 0.76 (0.50‒1.16) | 0.92 (0.67‒1.27) |
| **Sensitivity analysis by risk of bias items** |  |  |  |
| Studies with one or more unknown risk items omitted | 1.08 (0.79‒1.47) | NA | 1.11 (0.57‒2.16) |
| Studies with two or more unknown risk of bias items omitted | 1.08 (0.79‒1.47) | NA | 0.96 (0.65‒1.40) |
| Studies with three or more unknown risk of bias items omitted | 1.08 (0.79‒1.47) | NA | 0.96 (0.65‒1.40) |
| Conference abstracts omitted | 1.08 (0.79‒1.47) | NA | 0.96 (0.65‒1.40) |
| Low risk studies omitted | 1.01 (0.73‒1.38) | NA | 0.87 (0.60‒1.26) |
