# Supplementary material for: The efficacy and safety of S-1-based regimens in the first-line treatment of advanced gastric cancer: a systematic review and meta-analysis
Source: Gastric Cancer. 2016 Jan 11;19:696–712. doi: 10.1007/s10120-015-0587-8 (PMC4906062; doi:10.1007/s10120-015-0587-8)
Supplement: Supplementary file 6 — Supplementary material 6 (DOCX 15 kb) Table S2. Sensitivity analyses for S-1 combination therapy compared with S-1 monotherapy. Exploring heterogeneity by sensitivity analysis of omitting studies according to their risk of bias and subanalysis of study regions within Asia. The 95 % confidence intervals of China and Japan do not overlap and the point estimate (hazard ratio) of China was 0.30 lower compared with that of Japan. This may be a possible explanation for the heterogeneity in the main analysis. CI confidence interval, NA not available, RR risk ratio [file 10120_2015_587_MOESM6_ESM.docx]

**Supplementary Table S2. Sensitivity analyses for S-1 combination therapy compared to S-1 monotherapy.**

|  | **S-1 combination therapy vs S-1 monotherapy** | | |
| --- | --- | --- | --- |
|  | **OS HR (95%CI)** | **PFS HR (95%CI)** | **ORR RR (95%CI)** |
| **Full analysis set** | 0.76 (0.65‒0.90) | 0.68 (0.56‒0.82) | 1.51 (1.32‒1.74) |
| **Sensitivity analysis by risk of bias items** |  |  |  |
| Studies with one or more unknown risk items omitted | 0.82 (0.69‒0.98) | 0.57 (0.44‒0.73)* | 1.63 (1.27‒2.10) |
| Studies with two or more unknown risk of bias items omitted | 0.68 (0.55‒0.86) | 0.57 (0.44‒0.73)* | 1.63 (1.27‒2.10) |
| Studies with three or more unknown risk of bias items omitted | 0.73 (0.62‒0.87) | 0.65 (0.54‒0.79) | 1.57 (1.34‒1.84) |
| Conference reports omitted | 0.78 (0.69‒0.90) | 0.69 (0.56‒0.86) | 1.57 (1.34‒1.84) |
| Low risk studies omitted | 0.71 (0.54‒0.93) | 0.72 (0.59‒0.88) | 1.46 (1.24‒1.73) |
| **Study containing S-1 + Leucovorin omitted**** | 0.73 (0.62‒0.87) | 0.75 (0.64‒0.87) | 1.57 (1.36‒1.82) |
| **Region of Asian studies** |  |  |  |
| Studies conducted in China | 0.54 (0.41‒0.71) | 0.59 (0.42‒0.82) | 1.67 (1.22‒2.28) |
| Studies conducted in Japan | 0.83 (0.74‒0.94) | 0.71 (0.55‒0.92) | 1.48 (1.27‒1.73) |
| Studies conducted in Korea | 0.90 (0.67‒1.21) | NA | NA |

Notes:

*****Koizumi 2008 was the only study left over in this comparison.

** The comparison of S-1 + cisplatin versus S-1 + leucovorin in Yamaguchi 2014 was omitted.
