# Supplementary material for: The efficacy and safety of S-1-based regimens in the first-line treatment of advanced gastric cancer: a systematic review and meta-analysis
Source: Gastric Cancer. 2016 Jan 11;19:696–712. doi: 10.1007/s10120-015-0587-8 (PMC4906062; doi:10.1007/s10120-015-0587-8)
Supplement: Supplementary file 7 — Supplementary material 7 (DOCX 22 kb) Table S3. Toxicity results of S-1-based combination therapy compared with S-1 monotherapy. A risk ratio (RR) greater than 1 represents a beneficial effect for the experimental arm. CI confidence interval, N safety sample size, NA not available [file 10120_2015_587_MOESM7_ESM.docx]

**Table S3. Toxicity results of S-1 based combination therapy compared to S-1 monotherapy**

|  | **Grade 1-2** | | | | | | | | | **Grade 3-4** | | | | | | | | |
| --- | --- | --- | --- | --- | --- | --- | --- | --- | --- | --- | --- | --- | --- | --- | --- | --- | --- | --- |
|  | **S-1 combo** | | **S-1 alone** | | **Estimate** | | | **Heterogeneity** | | **S-1 combo** | | **S-1 alone** | | **Estimate** | | | **Heterogeneity** | |
|  | n | N | n | N | RR (95%CI) | *P* | Trials | I^2^ (%) | *P* | n | N | n | N | RR (95%CI) | *P* | Trials | I^2^ (%) | *P* |
| **Hematological** |  |  |  |  |  |  |  |  |  |  |  |  |  |  |  |  |  |  |
| Neutropenia | 172 | 486 | 175 | 492 | 1.00 (0.85-1.18) | 0.99 | 6 | 0 | 0.75 | 273 | 921 | 72 | 932 | 3.58 (2.67-4.80) | <0.001* | 9 | 23 | 0.24 |
| Leucopenia | 272 | 486 | 186 | 492 | 1.46 (1.28-1.67) | <0.001* | 6 | 0 | 0.55 | 137 | 921 | 28 | 932 | 4.17 (2.65-6.56) | <0.001* | 9 | 13 | 0.33 |
| Anemia | 180 | 438 | 137 | 445 | 1.32 (1.05-1.67) | 0.02* | 5 | 25 | 0.25 | 146 | 921 | 69 | 932 | 2.23 (1.46-3.40) | <0.001* | 9 | 44 | 0.07 |
| Thrombocytopenia | 96 | 391 | 51 | 398 | 1.84 (1.29-2.64) | <0.001* | 4 | 16 | 0.31 | 29 | 825 | 14 | 838 | 1.72 (0.54-5.53) | 0.36 | 7 | 53 | 0.04* |
| Lymphopenia | 15 | 95 | 4 | 94 | 3.67 (1.26-10.70) | 0.02* | 2 | 0 | 0.70 | 12 | 172 | 9 | 174 | 1.59 (0.39-6.45) | 0.51 | 3 | 45 | 0.16 |
| Febrile neutropenia | NA | NA | NA | NA | NA | NA | NA | NA | NA | 24 | 797 | 12 | 805 | 1.60 (0.61-4.20) | 0.34 | 7 | 30 | 0.20* |
| **Non-hematological** |  |  |  |  |  |  |  |  |  |  |  |  |  |  |  |  |  |  |
| Nausea | 281 | 486 | 207 | 492 | 1.24 (0.92-1.66) | 0.15 | 6 | 79 | <0.001* | 69 | 921 | 31 | 932 | 205 (1.26-3.33) | 0.004* | 9 | 12 | 0.33 |
| Vomiting | 135 | 391 | 94 | 398 | 1.50 (0.94-2.38) | 0.09 | 4 | 68 | 0.03* | 32 | 825 | 18 | 838 | 1.75 (0.98-3.15) | 0.06 | 7 | 0 | 0.86 |
| Diarrhea | 196 | 486 | 145 | 492 | 1.34 (1.13-1.59) | <0.001* | 6 | 0 | 0.64 | 59 | 921 | 39 | 932 | 1.53 (0.87-2.69) | 0.14 | 9 | 31 | 0.17 |
| Stomatitis | 136 | 486 | 137 | 492 | 0.97 (0.70-1.34) | 0.86 | 5 | 61 | 0.02* | 27 | 845 | 12 | 852 | 2.19 (1.13-4.26) | 0.02* | 8 | 0 | 0.86 |
| Anorexia | 267 | 486 | 196 | 492 | 1.35 (1.17-1.57) | <0.001* | 6 | 14 | 0.33 | 167 | 845 | 98 | 852 | 1.89 (1.19-3.00) | 0.007* | 8 | 65 | 0.005* |
| Fatigue | 270 | 486 | 199 | 539 | 1.35 (1.20-1.53) | <0.001* | 6 | 0 | 0.62 | 55 | 845 | 43 | 852 | 1.27 (0.86-1.88) | 0.23 | 8 | 0 | 0.93 |
| Hand foot syndrome | 30 | 391 | 33 | 398 | 0.92 (0.57-1.48) | 0.73 | 4 | 0 | 0.86 | NA | NA | NA | NA | NA | NA | NA | NA | NA |
| Sensory neuropathy | 58 | 283 | 14 | 285 | 3.56 (0.27-46.29) | 0.33 | 4 | 85 | <0.001* | 6 | 94 | 1 | 94 | 3.65 (0.58-22.82) | 0.17 | 2 | 0 | 0.42 |
| Alopecia | 87 | 155 | 13 | 160 | 6.91 (4.03-11.85) | <0.001* | 1 | NA | NA | NA | NA | NA | NA | NA | NA | NA | NA | NA |
| Rash | 45 | 236 | 43 | 238 | 1.06 (0.72-1.54) | 0.77 | 3 | 0 | 0.76 | 9 | 546 | 10 | 551 | 0.91 (0.36-2.32) | 0.85 | 4 | 0 | 0.69 |
| Lacrimation | 40 | 236 | 38 | 238 | 1.06 (0.71-1.60) | 0.76 | 3 | 0 | 0.74 | 1 | 236 | 2 | 238 | 0.70 (0.11-4.38) | 0.70 | 3 | 0 | 0.54 |
| Pigmentation | 163 | 391 | 165 | 398 | 1.01 (0.86-1.19) | 0.88 | 4 | 0 | 0.80 | NA | NA | NA | NA | NA | NA | NA | NA | NA |
| Serious adverse events | NA | NA | NA | NA | NA | NA | NA | NA | NA | 21 | 148 | 18 | 150 | 1.18 (0.66-2.13) | 0.58 | 1 | NA | NA |
| Toxicity related death | NA | NA | NA | NA | NA | NA | NA | NA | NA | 4 | 465 | 0 | 473 | 5.10 (0.60-43.50) | 0.14 | 2 | 0 | 0.99 |
